# Generation of new inhibitors of selected cytochrome P450 subtypes– *In silico* study

**DOI:** 10.1016/j.csbj.2022.10.005

**Published:** 2022-10-06

**Authors:** Tomasz Danel, Agnieszka Wojtuch, Sabina Podlewska

**Affiliations:** a Faculty of Mathematics and Computer Science, Jagiellonian University, 6 Łojasiewicza Street, 30-348 Kraków, Poland; b Maj Institute of Pharmacology, Polish Academy of Sciences, Department of Medicinal Chemistry, 31-343 Kraków, Smętna Street 12, Poland

**Keywords:** CYP450, New compounds generation, Docking, On-line platform, Explainability, CYP inhibitors, Graph neural networks, CYP, cytochrome P450, GNN, graph neural network, ML, machine learning, QSPR, quantitative structure-property relationship, DL, deep learning, DNNs, deep neural networks, RF, random forest, Morgan FP, Morgan fingerprint, SRD, sum of ranking differences, MSE, mean squared error

## Abstract

Physicochemical and pharmacokinetic compound profile has crucial impact on compound potency to become a future drug. Ligands with desired activity profile cannot be used for treatment if they are characterized by unfavourable physicochemical or ADMET properties. In the study, we consider metabolic stability and focus on selected subtypes of cytochrome P450 – proteins, which take part in the first phase of compound transformations in the organism. We develop a protocol for generation of new potential inhibitors of selected cytochrome isoforms. Its subsequent stages are composed of generation and assessment of new derivatives of known cytochrome inhibitors, docking and evaluation of the compound possible inhibition on the basis of the obtained ligand–protein complexes. Besides the library of new potential agents inhibiting particular cytochrome subtypes, we also prepare a graph neural network that predicts the change in activity for all modifications of the starting molecule. In addition, we perform a systematic statistical study on the influence of particular substitutions on the potential inhibition properties of generated compounds (both mono- and di-substitutions are considered), provide explanations of the inhibitory predictions and prepare an on-line visualization platform enabling manual inspection of the results. The developed methodology can greatly support the design of new cytochrome P450 inhibitors with the overarching goal of generation of new metabolically stable compounds. It enables instant evaluation of possible compound-cytochrome interactions and selection of ligands with the highest potential of possessing desired biological activity.

## Highlights

• A protocol for generation of new potential CYP inhibitors was developed.
• Evaluation scheme of generated compound libraries is provided.
• Innovative graph neural network model for substitution effect prediction was constructed.
• Visualization platform for manual results examination was prepared.
• Explanations of compound activity predictions are provided.

## Graphical abstract

**Figure:**
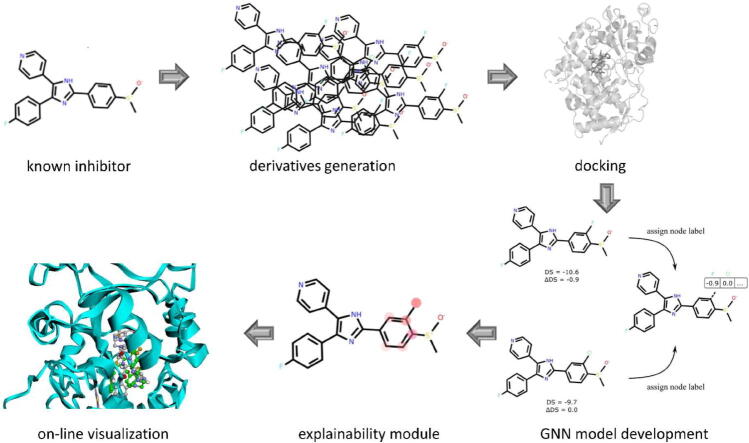

## 1 Introduction

Various computational strategies are an indispensable part of the drug design process [1], [2]. They support the development of new active compounds, as well as the optimization of their physicochemical and pharmacokinetic properties [3], [4], [5]. *In silico* methods used in the search for new active compounds can be divided into the *ligand-* [6] and *structure-based* [7] approaches. In the first case, the predictions are made based on the information of known ligands, both in terms of their activity and properties. On the other hand, in the case of the *structure-based* methods, it is the information about the target structure that is used to predict contacts between the potential ligands and target proteins.

To design an effective drug one must guarantee that after entering an organism it will have enough time to trigger a desired biological response. However, at the same time, the drug is constantly exposed to processes leading to its decomposition, which shorten its time of action and might also result in the formation of toxic products [8], [9], [10]. Unfortunately, biological processes occurring in the living organisms are very complex, and most often they are related to interactions with more than a single target. This makes metabolic stability one of the most difficult properties to evaluate using *in silico* methods. Nevertheless, both compound stability as well as other ADMET properties are extremely important for the potential compound success in drug design campaign, as even the most active compound will not pass to the subsequent stages of drug development pipeline if its ADMET properties are unfavourable.

Metabolic processes related to the final removal of xenobiotics from the organism can be divided into two main phases. In the first phase, which is the focal point of this study, the main role is played by cytochrome P450 (CYP) – a group of haemoprotein enzymes with monooxidase activity. There are almost 60 different CYP subtypes occurring in the human organism; however, some subtypes are involved to a much higher extent in the metabolic processes, such as CYP3A4, which is responsible for transformations of over 50 % of drugs [11], [12], [13].

When a compound comes into interaction with a particular CYP enzyme, it can slow down its transformation processes (inhibitors) or induce them (inducers). In this study, we concentrate on inhibition of selected CYP subtypes (CYP3A4, CYP2D6, CYP2C8, CYP2C9) and develop a methodology for generation of new inhibitors of these CYP proteins. As a starting point, we use known CYP inhibitors and modify their structure by adding selected chemical groups. The inhibition potency of the generated compounds is evaluated via docking and only the most potent compounds are finally returned. We use this database to perform a systematic analysis of the influence of particular substitutions on compound inhibition potency and provide the knowledge base for the design of new CYP inhibitors.

Furthermore, we use this database to develop a graph neural network (GNN) [14] that predicts the change in the compound CYP inhibition properties for all modifications of the starting molecule. Moreover, for each newly generated structure, we provide an explanation of the prediction of its inhibitory potency. It enables the indication of the structural moieties, which are most important for the particular prediction. Therefore, such explanations can be used to guide the further optimization of the compound structure in terms of its CYP inhibitory properties, especially when combined with the prediction of the inhibitory power given by the GNN model. In addition, baseline machine learning (ML) models for the binary prediction of the docking score change (either decrease or increase) upon particular substitution are developed (ML models for metabolic stability prediction expressed as half-lifetime have already been constructed by several research groups [15], [16], [17], [18]). It is worth noting, that it is an innovative approach of GNN application in computer-aided drug design, as it is the first time, when a compound graph is not treated as a whole, but particular graph vertices constitute basis for docking score predictions and assist in compound optimization.

**Fig. 1:**
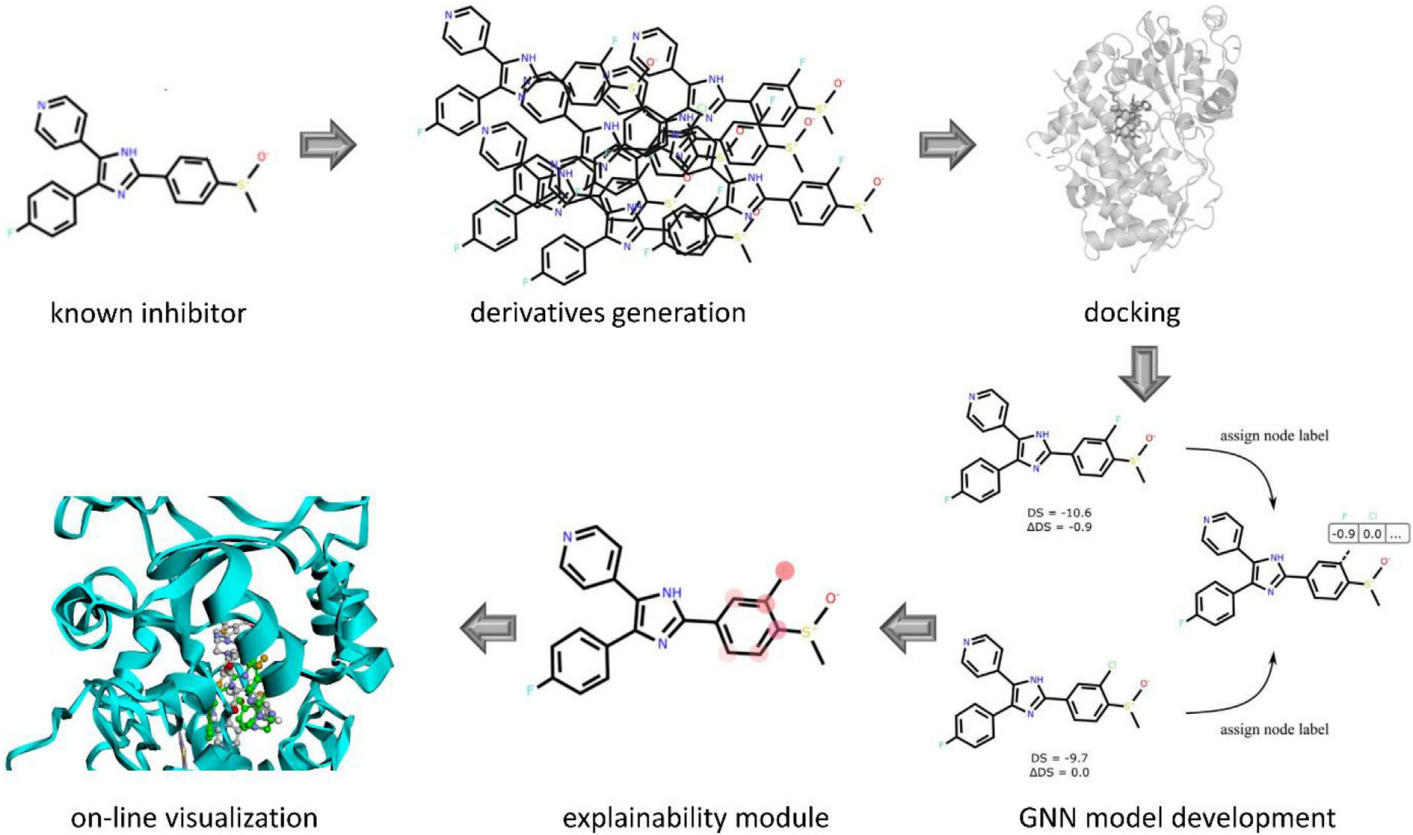
Summary of the main tasks carried out within the study.

Finally, we prepared an on-line visualization platform, where users can manually compare the compound poses in the respective CYP binding site, examine interactions and propose their own structural modifications (https://gmum.github.io/cyp-inhibitors/). All experiments carried out in the study can be reproduced for any target using the provided scripts (https://github.com/gmum/cyp-inhibitors). The library of newly generated potential CYP inhibitors is shared in the Supplementary Data. The visualization of the main aspects of the presented study is presented in Fig. 1.

A series of approaches to quantitative structure–property relationship (QSPR) tasks has already been proposed [19], [20], [21], [22], [23], [24], [25], [26], [27], [28], which are continuously evolving together with the development of new algorithms. GNNs used in the study have also already entered the field of computer-aided drug design, and they have been utilised for example in QSPR-related tasks due to their input being suitable to represent molecules and because of their superior performance [29], [30]. Wang *et al.* [31] trained GNNs to predict pIC50 values of JAK inhibitors, while classification of molecules as inhibitors or non-inhibitors of selected CYP450 isoforms was done by Wu *et al.* [32] who used ML and deep learning (DL) models or Li *et al.* [33] who utilised single- and multitask deep neural networks (DNNs).

In our study, we use GNNs to predict the exact value of docking score of a series of compound derivatives. The problem of docking score prediction in the literature was reported *e.g.* by Jastrzębski *et al.* [34] who concentrated on several GPCRs and selected CYPs and utilised GNNs or Ton *et al*. [35] who focused on SARS-CoV-2 main protease (Mpro) and used DNNs. Here, we direct our attention to selected CYP450 isoforms and employ GNNs to predict docking score change for all modifications of the input compound. This is different from the discussed work by that we predict a change in the docking score instead of the score itself, and, more importantly, because the prediction is made not for the input compound itself but for all of its modifications. We believe that such a construction of the predictive model is best suited for compound optimisation task because there is no need for manual definition of possible modifications and moreover, predictions for all of them are calculated and returned all at once.

## 2 Methods

### 2.1 Datasets preparation and compounds enumeration

**Fig. 2:**
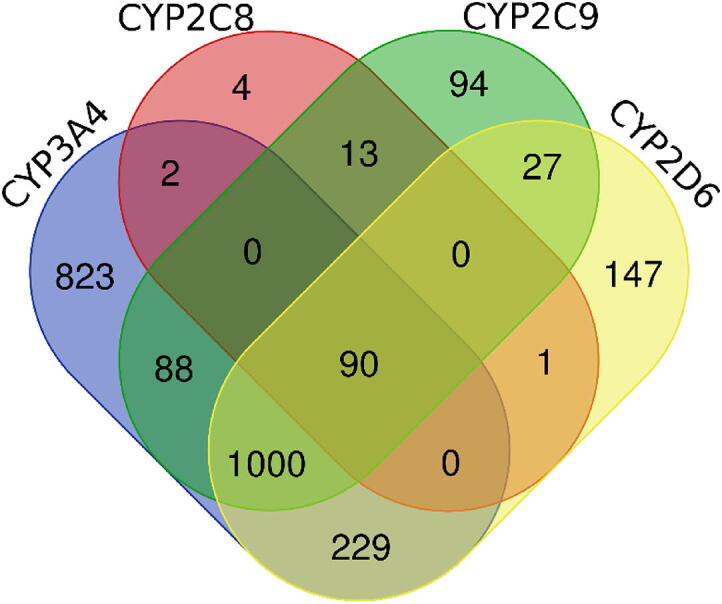
Intersection between the considered datasets (Venn diagram generated with the use of https://bioinformatics.psb.ugent.be/webtools/Venn/).

Compounds with known inhibition potency on the considered CYP subtypes (CYP3A4, CYP2D6, CYP2C8, and CYP2C9) were fetched from the ChEMBL database, version 27 [36] (the intersection between particular datasets is presented in Fig. 2). We filter out all records which do not refer to the Standard Type: Inhibition and Standard Units: % and the following number of data points remain: CYP3A4: 1900; CYP2D6: 1326; CYP2C8: 101; CYP2C9: 1000.

Then, the set of new potential CYP inhibitors is formed by addition of a respective substituent to the initial compound structure. The list of chemical fragments added is as follows: F, Cl, Br, I, C, C(C)C, CC, C(=O)O, O, OC, COC, CO, C(=O)C, N, S.

### 2.2 Crystal structures characterization and docking

All compounds (both these initially downloaded from ChEMBL database and the newly formed ones) are docked to the available CYP crystal structures. In all cases, we use two types of crystal structures: free and with inhibitor co-crystallized (Table 1).

**Table 1:** Crystal structures of CYP subtypes used in the study (crystal resolution is provided in brackets).

CYP subtype	CYP3A4	CYP2C8	CYP2C9	CYP2D6
With inhibitor	1W0G [37] (2.73 Å)	2NNI [38] (2.80 Å)	4NZ2 [40] (2.45 Å)	3QM4 [42] (2.85 Å)
Free	1W0E [37] (2.80 Å)	1PQ2 [39] (2.70 Å)	1OG2 [41] (2.60 Å)	2F9Q [43] (3.00 Å)

The docking is carried out in Smina [44] using the default settings and the Vina scoring function. We validate the docking via examination of the docking poses obtained for the co-crystallized inhibitors (Fig. S1). The proteins are cleaned by removing all non-protein atoms, excluding heme (the cytochromes cofactor). The information provided by docking (the docking score value) is used to create a new dataset that describes the change in the docking score for modifications of the starting molecule. The dataset obtained for considered CYP subtypes is available in Supplementary Data, and the code for generation of derivatives of ligands of any target is available on the GitHub repository (https://github.com/gmum/cyp-inhibitors).

### 2.3 GNN model

We use the dataset consisting of the information from docking (the docking score value) to train convolutional GNNs [14]. The task is defined as node regression, and the models are intended to predict an exact change in the docking score for each possible modification of the input compound. It is worth emphasizing that the change in the docking score for all possible modifications of the original compound is computed in a single pass. Such an approach is much more effective than making a separate calculation for each possible modification.

**Fig. 3:**
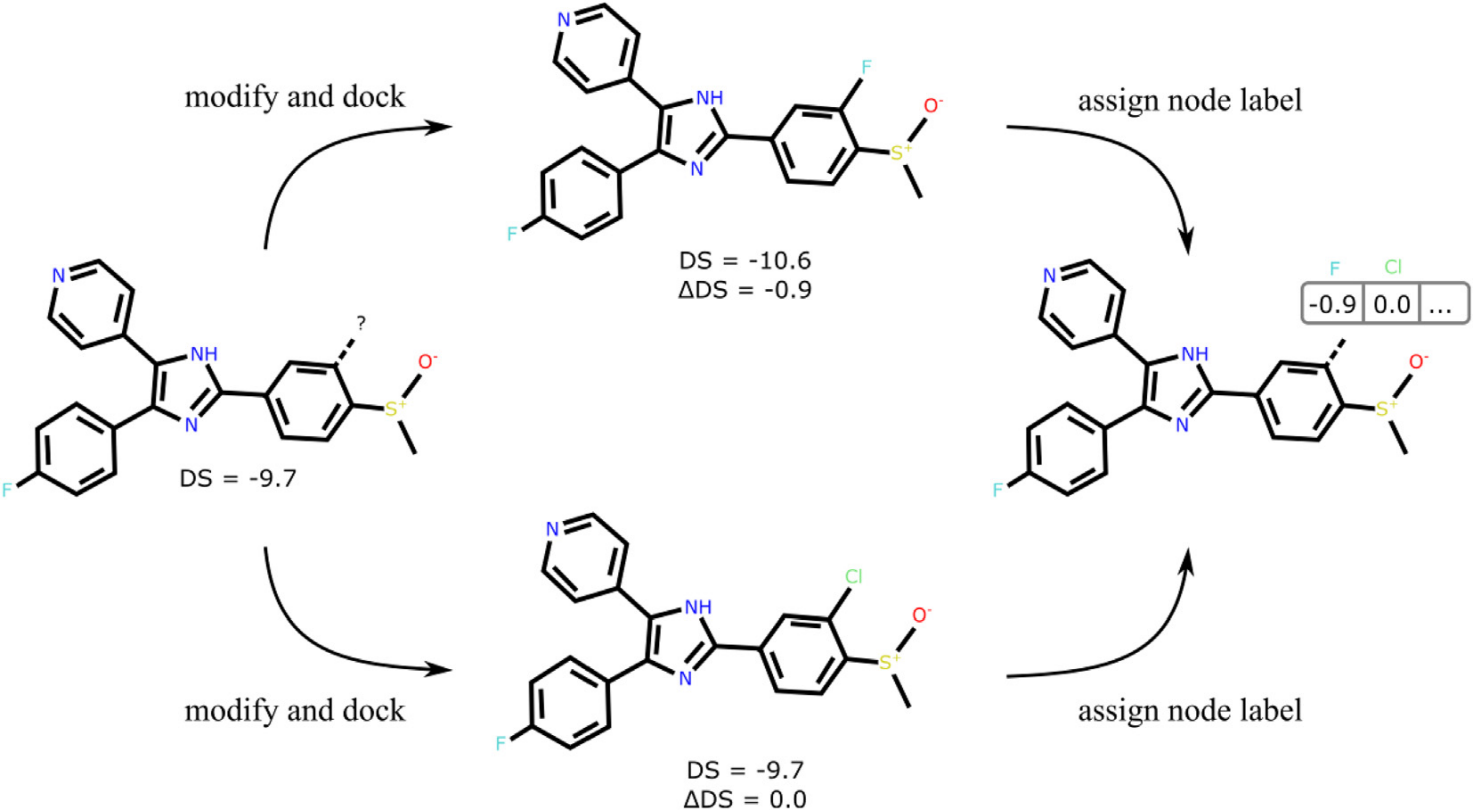
The overview of the process of assigning labels to graph nodes. On the left, an exemplary compound is shown, and the substitutions are made in the position marked with the question mark symbol. After substituting this atom, the modified compounds are docked, and the difference ΔDS between the docking score of the modified and original compound is calculated. Next, the differences for each substitution are assigned to the vector label for the given node, as depicted on the right. The process is repeated for all ring atoms in the original compound and for all the defined substitution groups. If any of the substitutions is not possible (*e.g.* due to valency constraints), a null value is assigned in the label vector, and this value is omitted in the training.

In the node regression task, the label for each node is a vector of changes in the docking score value for all substitutions used in this atom (Fig. 3). If an atom cannot be substituted, the corresponding position in the vector remains empty and is not used neither for training nor for testing.

The GNNs consist of 3 or 5 convolutional layers with the hidden representation size of 256. We use both classical convolutional layers proposed by Kipf *et al.* [14] and graph attention layers proposed by Veličković *et al.* [45] The convolutional layers are followed by one or two linear layers. All models use skip connections, BatchNorm [46] and dropout of 0.2 or 0.5. [47] All models are trained for 200 epochs with Adam, a learning rate of 0.01 or 0.001, batch size 256, and ReduceLROnPlateau scheduler with patience equal to 10. All models use weight decay of 0.0005 or no weight decay at all. As a training objective, we use masked MSE loss, that is MSE loss which ignores errors for substitutions that are not present in the training data. For each CYP subtype we train 64 different architectures using fivefold cross-validation to choose the best hyperparameters. The final model is evaluated using a held-out test set.

The molecules are represented using a graph molecular representation with the following atom features: atom type, the number of implicit hydrogens, the number of heavy-atom neighbours, formal charge, ring inclusion, and aromaticity. The resulting length of atom representation is 42. The information about bond features is not included.

### 2.4 ML reference models

As a reference, we develop models for the prediction of the direction of docking score change (increase or decrease). To this end, we use: a baseline approach, Random Forest (RF) [48], a GNN [14] and a GNN with a dummy node [49]. The summary of these models is presented in Table 2.

**Table 2:** Summary of the reference models.

Model	Representation	Description
Baseline	–	Returns the majority label
RandomForest	MorganFP	Predicts docking scores separately for each substitution
GNN	Graph representation	Node regression

As a baseline approach we use a model that assigns the most prevalent label in the dataset to each compound. This baseline shows the ratio between positive and negative classes in the dataset.

RF makes separate predictions of a docking score of the original and the modified compound. These predictions are compared to assign the change in the docking score. The molecules are represented with Morgan fingerprints (Morgan FP) [50], [51] with radius 2 and 1024 bit-length.

Both GNN models use graph molecular representation with the same atom features as previously. Here, the problem is again formulated as a node regression task – the docking score change is predicted for each atom of the original compound and for each possible modification in a single pass.

GNN is a classical graph convolutional model introduced by Kipf *et al*. [14], while GNN-dummy is an extension of the GNN model, which includes additional nodes, called dummy nodes, in the molecular graph [49]. These nodes are connected to all the other nodes, and their purpose is the aggregation of the signal from the whole molecular graph in each graph convolutional layer. This way the perception field of the convolution is artificially extended beyond the atom neighbours.

### 2.5 Explainability

Explainability is a quickly growing field of ML [52], [53], [54]. Its techniques aim to elucidate inner workings of black box models. In this work, we use an explainability technique, called saliency maps, in order to provide information about the influence of particular atoms on the predictions.

Saliency maps are a visualisation technique introduced by Simonyan *et al.* [55] They explain the predictions of the model by an analysis of its partial derivatives and can be seen as a sensitivity analysis technique [56]. The main idea behind this approach is that derivatives can be seen as a measure of how sensitive is the function's output with respect to its input. Formally, a saliency map is calculated by measuring a length of a vector of positive partial derivatives: $\|ReLU\left(\frac{\delta y}{\delta x}\right)\|$, where $\frac{\delta y}{\delta x}$ is the derivative over the output with respect to input, ReLUx:=max0,x, and ‖∙‖ is the Euclidean norm.

In the classical approach, only the positive gradients contribute to the final result. Apart from this, we also investigate the influence of negative partial derivatives ($\|ReLU\left(\frac{-\delta y}{\delta x}\right)\|$). This allows us to compare the influence of positive and negative partial derivatives which we illustrate by calculating a difference between classical and negative saliency maps and call this approach positive–negative saliency maps.

### 2.6 Visualization platform

In order to enable visual comparison of the obtained docking poses, we prepared an on-line visualization platform (https://gmum.github.io/cyp-inhibitors/). It enables manual confrontation of the docking poses of original compound and its derivatives, constituting a great support during interpretation of the docking score changes occurring upon substitution. In the platform, we incorporate results only for the top 100 compounds (in terms of their docking score value), while the docking poses for all derivatives obtained in the study are available at https://github.com/gmum/cyp-inhibitors/tree/main/data/poses.

## 3 Results and discussion

### 3.1 ChEMBL data

**Fig. 4:**
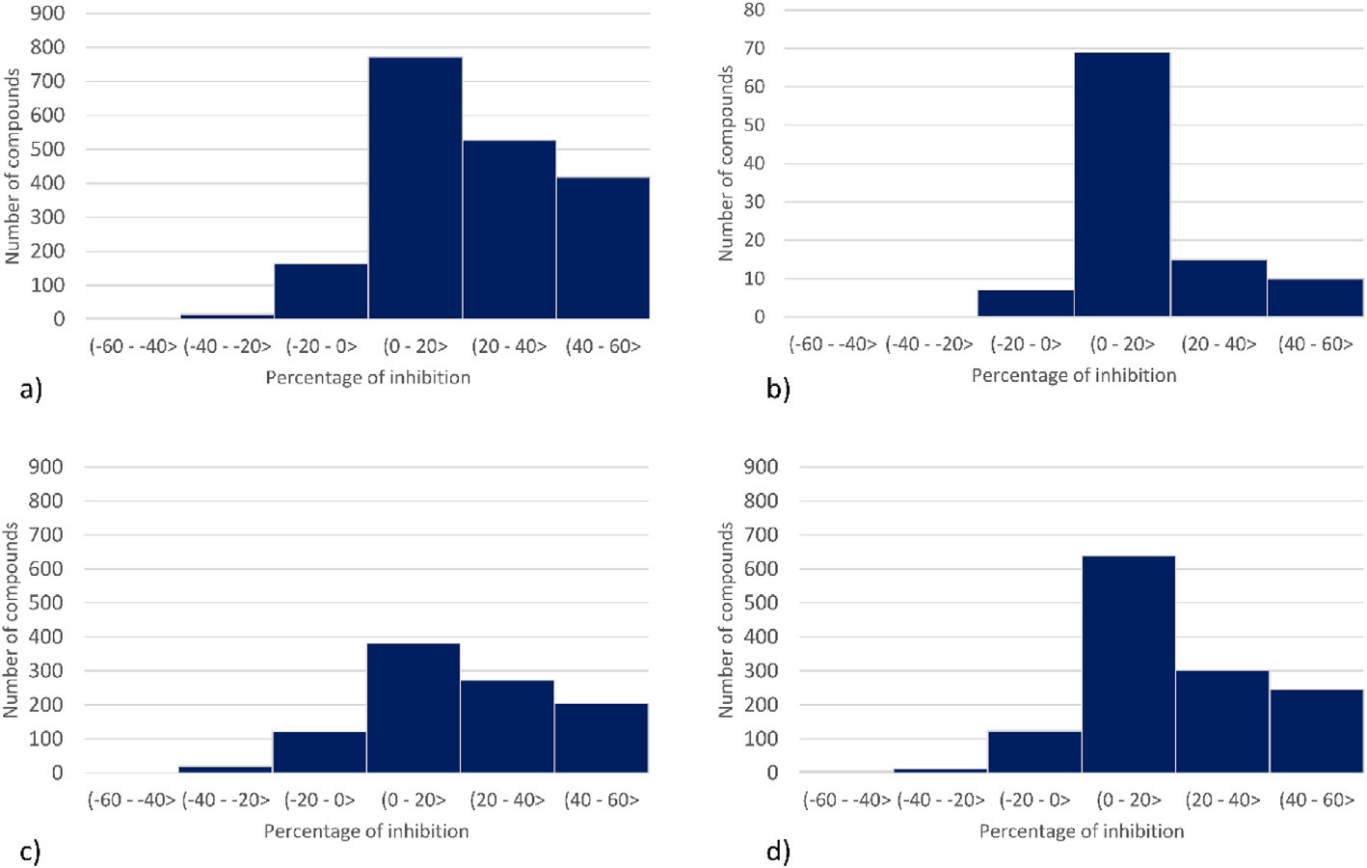
Histogram of percentage of inhibition of CYP by a compound for datasets for a) CYP3A4, b) CYP2C8, c) CYP2C9, d) CYP2D6.

The distribution of data used in the study is presented in Fig. 4. It shows that the distribution of the percentage of inhibition is similar for all examined CYP subtypes. The highest number of compounds fall in the range of subtle CYP inhibition (between 0 and 20 %), and the number of compounds from the subsequent inhibition ranges (20–40 % and 40–60 %) gradually decreases. In each case, there is also a small number of compounds which appear not to have the ability to inhibit CYPs (with inhibition percentage between −20 – 0%) and for CYP3A4, CYP2C9, and CYP2D6, there also exist several compounds that appear to be inducers with the reported CYP inhibition ability between −40 – −20 %.

**Fig. 5:**
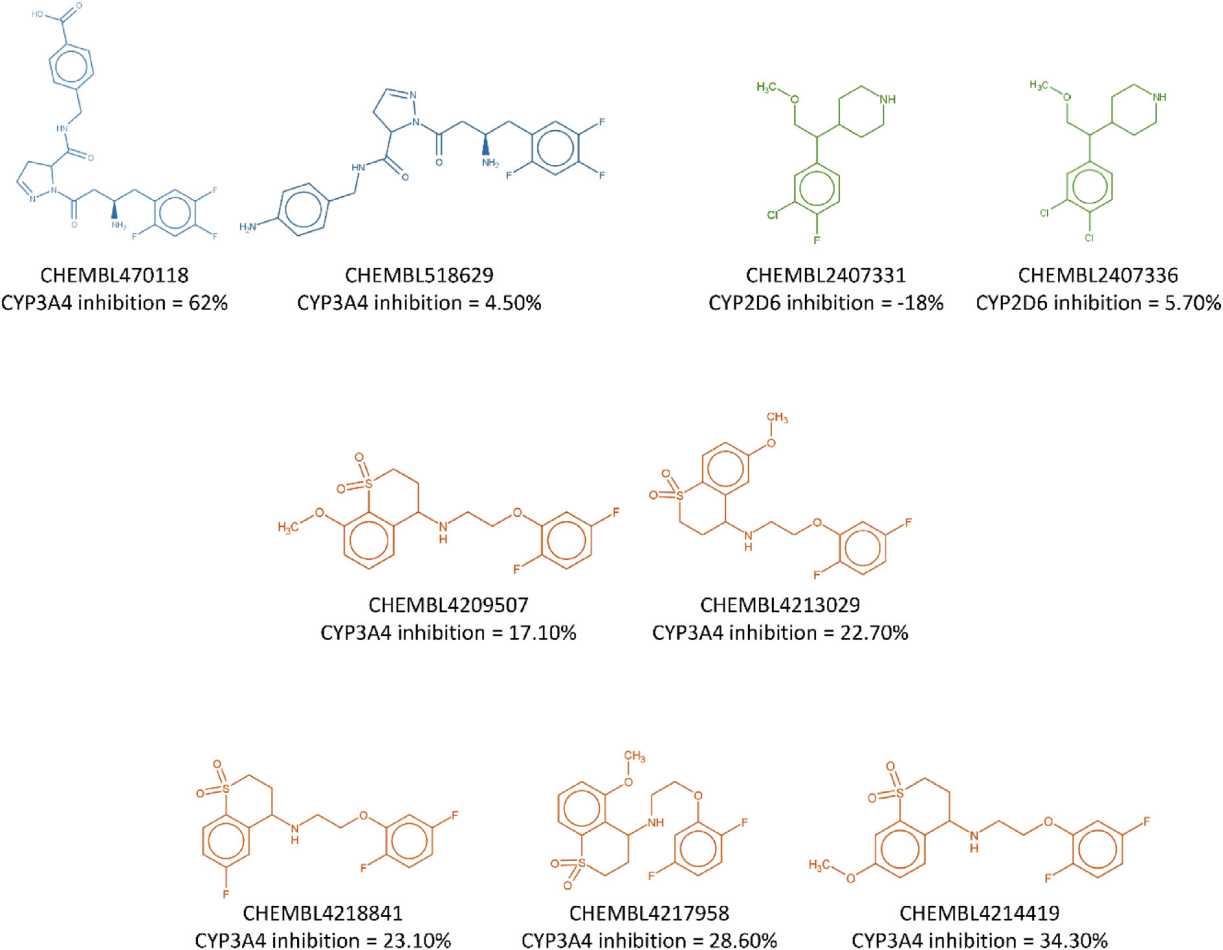
Examples of CYP inhibitors with different substituents present in the ChEMBL database.

Additionally, we examine the influence of particular substitutions on the CYP inhibition present in the ChEMBL data. For this particular analysis we do not use the docking results and narrow down to only these modifications that are present in the ChEMBL database. Selected examples are shown in Fig. 5.

In the case of compounds CHEMBL470118 and CHEMBL518629 (first row), where the carboxyl group is exchanged into the primary amine, the significant change in the CYP3A4 inhibitory potency is observed (62 % *vs* 4.5 %, respectively). On the other hand, replacement of fluorine atom by the chlorine results in the preservation of high inhibitory activity of CHEMBL2407331 and CHEMBL2407336 (first row). The series of CYP3A4 inhibitors varies in the position of methoxy group (in one case, it is replaced by the fluorine atom) and difluorophenyl attachment point; however, the variations in the CYP3A4 inhibitory potency are not very high – from 17 % for CHEMBL4209507 to 34 % of CHEMBL4214419 (middle and bottom row).

### 3.2 Analysis of the influence of particular substituents on docking score – monosubstitution

**Fig. 6:**
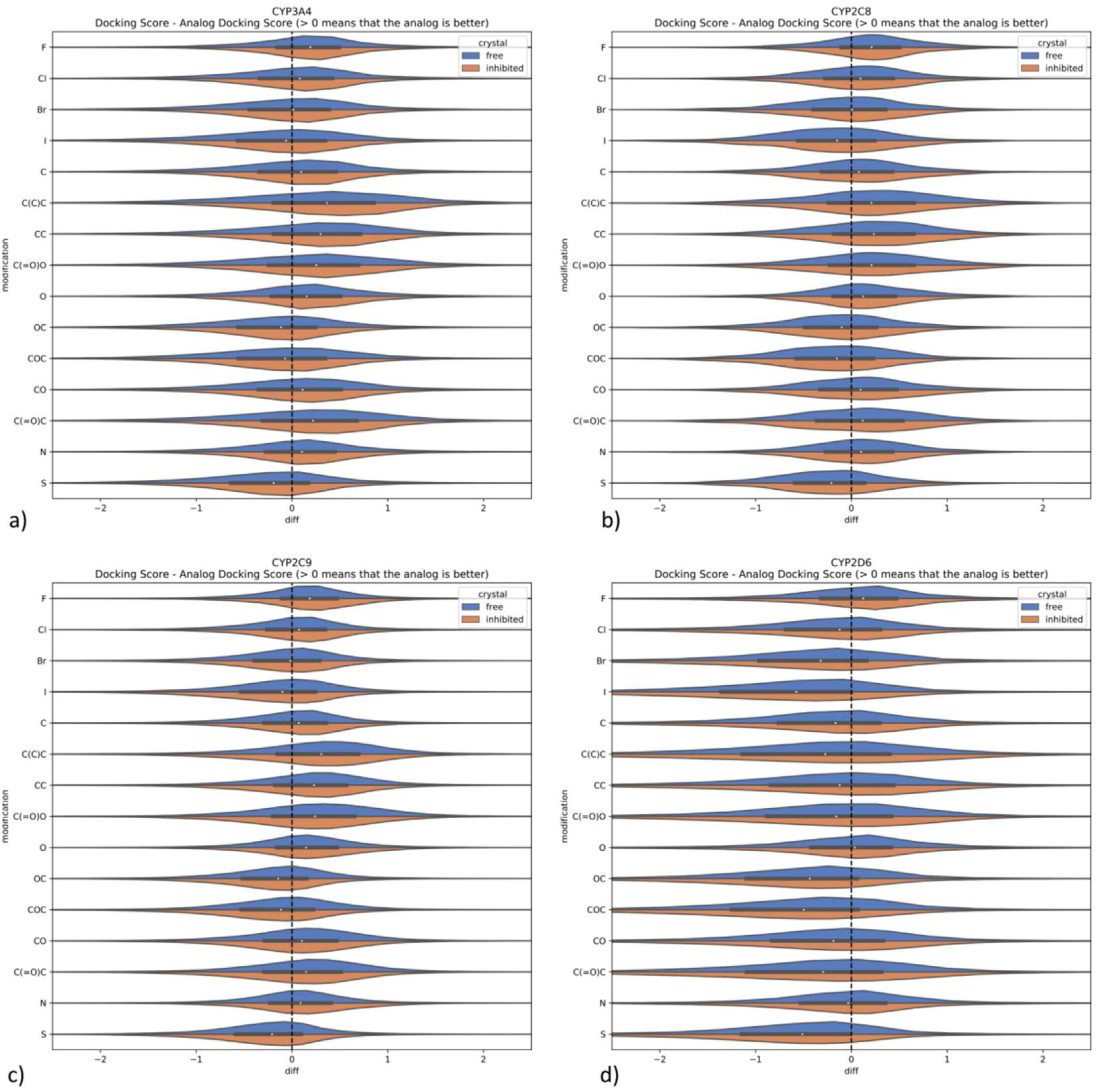
Changes in the docking score values for single substitution for a) CYP3A4, b) CYP2C8, c) CYP2C9, d) CYP2D6.

We dock all compounds (both original and the derivatives) to the respective CYP crystal structures and determine the docking scores of the obtained ligand–protein complexes. The differences in the docking scores between the original compounds and their derivatives are presented in Fig. 6.

The first observation is that the tendencies for each substituent are similar for free and for inhibited crystal structures – blue and orange parts of charts in Fig. 6 are similarly distributed. However, there is a variation between different substituents. In general, the addition of a halogen leads to an improvement of the docking score – this refers mainly to the fluorine and chlorine substitutions, which lead to more effective CYP inhibitors in comparison to the starting compounds (when docking score-based evaluation is taken into account). On the other hand, bromine and iodine substitutions are much less effective in improving the CYP inhibitors docking scores, and for CYP2C8 and CYP2D6 they even worsen the docking score values. Likewise, the addition of sulphur, OC or COC substituents always leads to the worsening of docking scores.

Despite similar distribution of general tendencies of docking score differences observed for free and inhibited crystal structures, examination of the results from the perspective of particular compound reveals that the outcome is in fact influenced by the type of the crystal structure (detailed analysis is present in Fig. S2).

**Fig. 7:**
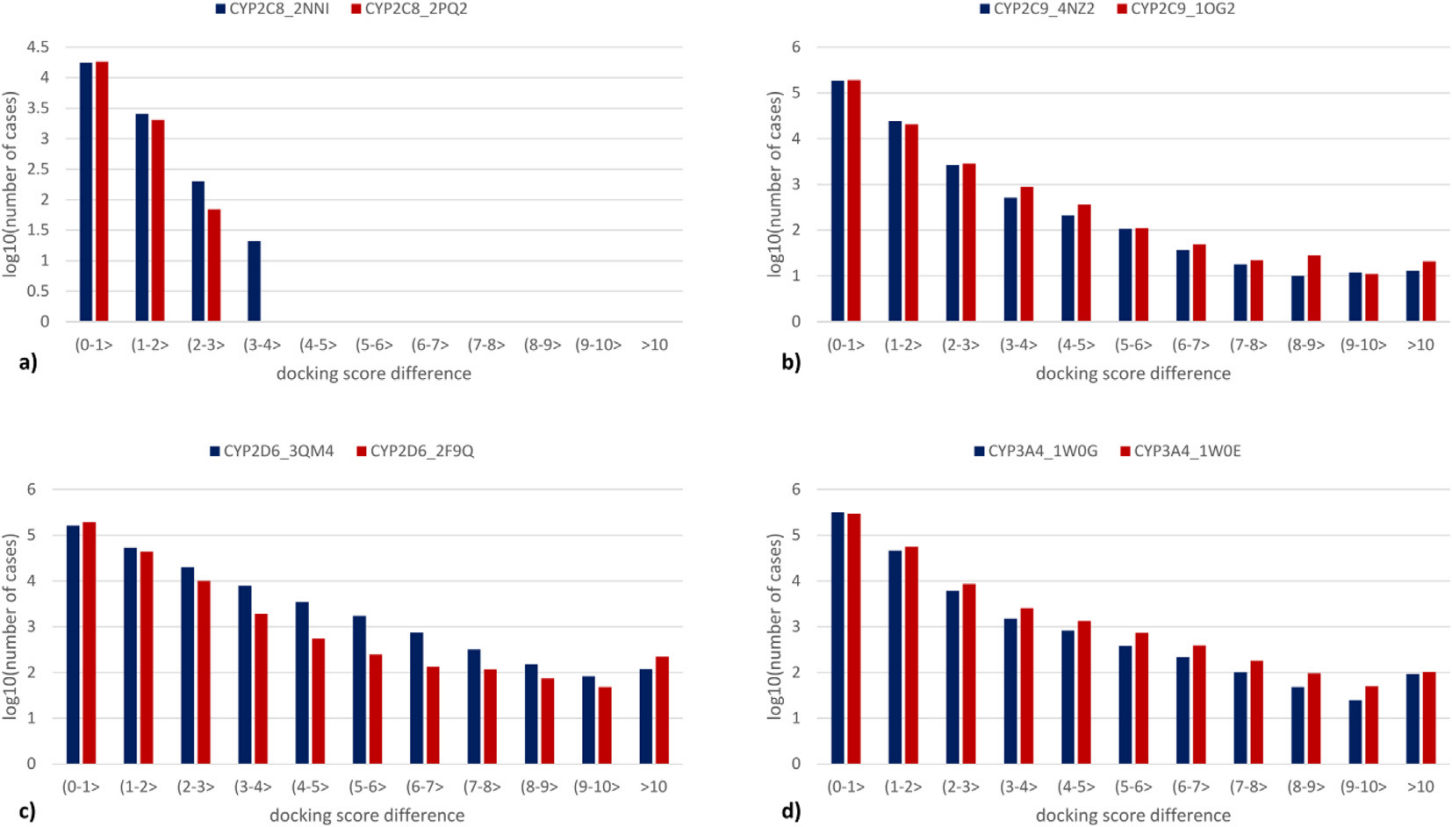
Histograms of the changes in the docking scores upon substitution in comparison to the original compound for a) CYP2C8, b) CYP2C9, c) CYP2D6, d) CYP3A4. For better clarity, the logarithmic scale for the number of cases is applied. (For interpretation of the references to color in this figure legend, the reader is referred to the web version of this article.)

In Fig. 7, we present histograms of changes in the docking scores (detailed information is available in the Supplementary Data). For a great majority of cases, changes in the docking score are below 1, although higher values are also observed. The fraction of compound poses with docking score difference between 1 and 2 is similar for all of the considered CYP subtypes. For CYP2C8, there are no poses with docking score change higher than 4 upon substitution. On the other hand, for all other CYP subtypes, there are variations in the docking score up to over 10, although they refer to less than 1 % of the total number of docking poses. To sum up, in some cases even a single substitution can lead to a huge difference in the docking score value; however, in most cases this difference is negligible.

### 3.3 Di-substitution experiments

**Fig. 8:**
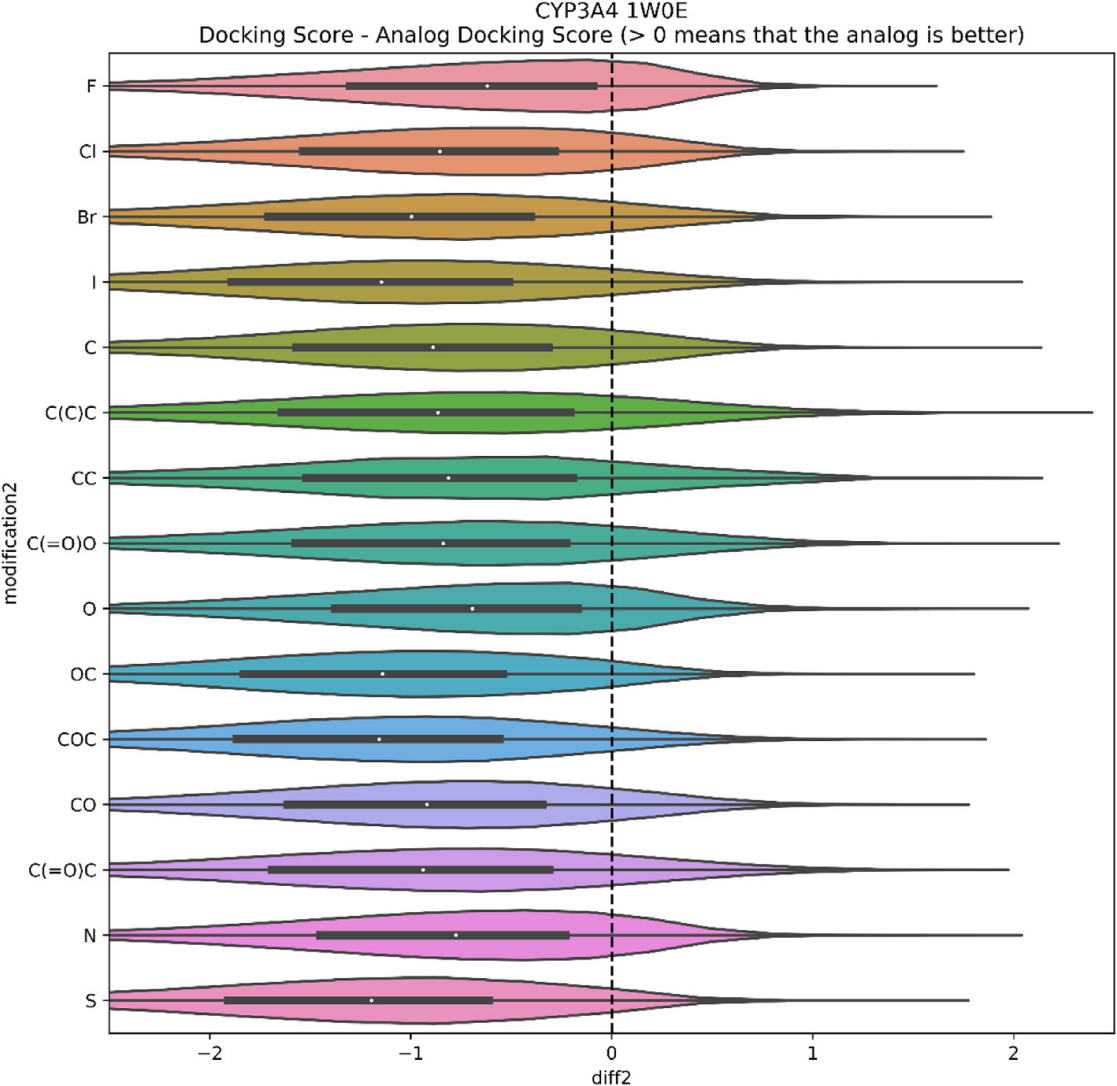
Changes in the docking score values between single and double substitution for CYP3A4 inhibitors.

To examine the possibilities offered by modifying an input molecule in more than a single place, we carry out an analysis of di-substituted compounds. A visualization of differences between the double and single substitutions is shown in Fig. 8. It is visible that in each case, the single substitution is preferred over the double one. The analysis is based on the averaged difference in docking scores regardless of the position of substitution.

### 3.4 Predictive performance of GNNs and ML models

To put the predictive performance of GNNs in context, we develop ML models with the aim of predicting the direction of the docking score change, which is a simpler task then predicting its exact value. The performance of the models is thoroughly examined using accuracy and mean squared error (MSE), which are calculated on both validation and held-out test sets. The evaluation on the held-out test set can be considered as a simulation of the real application of the constructed protocol to evaluate novel compounds (*e.g.* during the virtual screening procedure where compounds covering broad chemical space undergo evaluation), as it constitutes a fully external dataset. We use two approaches to divide data into train and held-out test sets – random selection and time-based selection (on the basis of the record list from the ChEMBL database).

The prediction accuracies and MSE values are gathered in Table 3, Table 4, Table 5, Table 6 (baseline assigns label of the majority class to all samples, RF accuracies are determined only for time split). The values shown in Table 3 and Table 4 indicate high variations in the prediction accuracy depending on the model. In almost all cases, GNN appears to be the most effective model with prediction accuracy between ∼0.58 to 0.67. The performance of both GNN and GNN-dummy models in the prediction of correct direction of change in the docking score upon substitution is better than the baseline by up to ∼0.15, which justifies the application of the developed approach. The performance of RF is close to the baseline, indicating its inability to correctly learn the patterns in the data and justifying the necessity of developing more sophisticated models, such as GNNs. In addition, we observe no significant improvement in the performance of the GNN-dummy model over its classical GNN counterpart, which may indicate either ineffective aggregation of the molecular graph by the dummy node or a strong correlation between the docking score change and local features of the chemical structures in the GNN model. The performance dependencies are similar for both types of crystal structures – free (Table 3) and co-crystallized with inhibitor (Table 4). GNNs and GNN-dummy models are also the most effective methods when the compounds are ranked on the basis of the predicted docking score values, which is indicated by the lowest MSE out of all of the compared approaches (Table 5, Table 6).

**Table 3:** Accuracy of the prediction of the docking score change (increase or decrease) after compound modification for crystal structures with CYP inhibitor co-crystallized for validation and held-out test sets. The best predictions for the particular crystal are depicted in bold.

Accuracy on validation test set (random split)				
Model	CYP3A4 (1W0G)	CYP2C8 (2NNI)	CYP2C9 (4NZ2)	CYP2D6 (3QM4)
Baseline	0.5725 ± 0.0095	0.5365 ± 0.0095	0.5185 ± 0.0327	0.6162 ± 0.0137
GNN	0.6287 ± 0.0010	**0.6115 ± 0.0140**	**0.6344 ± 0.0030**	**0.6545 ± 0.0090**
GNN-dummy	**0.6289 ± 0.0010**	0.6018 ± 0.0161	0.6324 ± 0.0041	0.6534 ± 0.0074

Accuracy on validation test set (time split)				
Model	CYP3A4 (1W0G)	CYP2C8 (2NNI)	CYP2C9 (4NZ2)	CYP2D6 (3QM4)

Baseline	0.5201 + 0.0112	0.5331 + 0.0188	0.4779 + 0.0183	0.5611 + 0.0190
RF	0.5114 + 0.0063	0.5371 + 0.0090	0.5147 + 0.0142	0.5697 + 0.0155
GNN	0.6487 + 0.0117	0.6285 + 0.0223	**0.6624 + 0.0055**	0.6680 + 0.0117
GNN-dummy	**0.6515 + 0.0102**	**0.6323 + 0.0225**	0.6587 + 0.0091	**0.6721 + 0.0152**

Accuracy on held-out test set (random split)				
Model	CYP3A4 (1W0G)	CYP2C8 (2NNI)	CYP2C9 (4NZ2)	CYP2D6 (3QM4)

Baseline	0.5480 + 0.0000	0.5760 + 0.0000	0.5048 + 0.0236	0.5952 + 0.0000
GNN	**0.6248 + 0.0021**	**0.6111 + 0.0067**	**0.6351 + 0.0023**	0.6395 + 0.0027
GNN-dummy	0.6196 + 0.0023	0.6071 + 0.0091	0.6308 + 0.0030	**0.6415 + 0.0034**

Accuracy on held-out test set (time split)				
Model	CYP3A4 (1W0G)	CYP2C8 (2NNI)	CYP2C9 (4NZ2)	CYP2D6 (3QM4)

Baseline	0.5946 + 0.0000	0.5426 + 0.0568	0.5088 + 0.0432	0.6221 + 0.0000
RF	0.5072 + 0.0098	0.5262 + 0.0264	0.5151 + 0.0114	0.5942 + 0.0058
GNN	0.6246 + 0.0020	0.5859 + 0.0109	0.6196 + 0.0057	**0.6433 + 0.0056**
GNN-dummy	**0.6272 + 0.0037**	**0.5972 + 0.0208**	**0.6224 + 0.0126**	0.6411 + 0.0068

**Table 4:** Accuracy of the prediction of the docking score change (increase or decrease) after compound modification for free enzymes for validation and held-out test set. The best predictions for the particular crystal are depicted in bold.

Accuracy on validation test set (random split)				
Model	CYP3A4 (1W0E)	CYP2C8 (1PQ2)	CYP2C9 (1OG2)	CYP2D6 (2F9Q)
Baseline	0.4819 + 0.0417	0.5407 + 0.0518	0.5263 + 0.0486	0.6038 + 0.0126
GNN	**0.6147 + 0.0113**	0.6275 + 0.0121	**0.6199 + 0.0111**	0.6496 + 0.0084
GNN-dummy	0.6097 + 0.0203	**0.6381 + 0.0152**	0.6198 + 0.0104	**0.6502 + 0.0081**

Accuracy on validation test set (time split)				
Model	CYP3A4 (1W0E)	CYP2C8 (1PQ2)	CYP2C9 (1OG2)	CYP2D6 (2F9Q)

Baseline	0.4423 + 0.0289	0.5012 + 0.0327	0.5067 + 0.0097	0.5546 + 0.0172
RF	0.5117 + 0.0153	0.5271 + 0.0257	0.5021 + 0.0092	0.5784 + 0.0105
GNN	0.6376 + 0.0152	0.6496 + 0.0091	0.6451 + 0.0109	0.6766 + 0.0083
GNN-dummy	**0.6408 + 0.0098**	**0.6565 + 0.0251**	**0.6476 + 0.0124**	**0.6781 + 0.0077**

Accuracy on held-out test set (random split)				
Model	CYP3A4 (1W0E)	CYP2C8 (1PQ2)	CYP2C9 (1OG2)	CYP2D6 (2F9Q)

Baseline	0.4766 + 0.0312	0.5150 + 0.0200	0.5214 + 0.1049	0.6148 + 0.0000
GNN	**0.6219 + 0.0126**	**0.5801 + 0.0054**	**0.6193 + 0.0073**	0.6538 + 0.0031
GNN-dummy	0.6067 + 0.0172	0.5647 + 0.0103	0.6067 + 0.0108	**0.6551 + 0.0033**

Accuracy on held-out test set (time split)				
Model	CYP3A4 (1W0E)	CYP2C8 (1PQ2)	CYP2C9 (1OG2)	CYP2D6 (2F9Q)

Baseline	0.4454 + 0.0728	0.5176 + 0.0235	0.5962 + 0.0000	0.5930 + 0.0000
RF	0.5034 + 0.0078	0.5647 + 0.0171	0.4940 + 0.0093	0.5799 + 0.0081
GNN	0.6076 + 0.0107	**0.6018 + 0.0045**	0.6233 + 0.0047	**0.6496 + 0.0040**
GNN-dummy	**0.6120 + 0.0045**	0.5843 + 0.0153	**0.6280 + 0.0064**	0.6458 + 0.0067

**Table 5:** MSE values obtained for compound ranking on the basis of the docking score change for crystal structures with CYP inhibitor co-crystallized for validation and held-out test sets. The lowest MSE values (referring to the lowest error and highest prediction accuracy) are depicted in bold.

MSE ranking on validation test set (random split)				
Model	CYP3A4 (1W0G)	CYP2C8 (2NNI)	CYP2C9 (4NZ2)	CYP2D6 (3QM4)
Baseline	0.6874 + 0.0805	0.4716 + 0.0580	0.5244 + 0.0533	2.0553 + 0.1301
GNN	0.6335 + 0.0778	**0.4349 + 0.0629**	**0.4607 + 0.0531**	1.8214 + 0.1069
GNN-dummy	**0.6309 + 0.0880**	0.4457 + 0.0602	0.4612 + 0.0526	**1.8059 + 0.1245**

MSE ranking on validation test set (time split)				
Model	CYP3A4 (1W0G)	CYP2C8 (2NNI)	CYP2C9 (4NZ2)	CYP2D6 (3QM4)

Baseline	0.7208 + 0.2266	0.4335 + 0.0720	0.5152 + 0.1331	1.9662 + 0.4721
GNN	**0.6814 + 0.2320**	**0.4134 + 0.0896**	**0.4651 + 0.1277**	1.8110 + 0.4353
GNN-dummy	0.6817 + 0.2311	0.4173 + 0.0823	0.4660 + 0.1282	**1.8023 + 0.4301**

MSE ranking on held-out test set (random split)				
Model	CYP3A4 (1W0G)	CYP2C8 (2NNI)	CYP2C9 (4NZ2)	CYP2D6 (3QM4)

Baseline	1.1960 + 0.0008	0.4728 + 0.0037	0.7681 + 0.0018	1.9295 + 0.0077
GNN	**1.1310 + 0.0139**	**0.4382 + 0.0084**	**0.6925 + 0.0051**	1.7523 + 0.0148
GNN-dummy	1.1756 + 0.0413	0.4439 + 0.0068	0.6956 + 0.0071	**1.7417 + 0.0294**

MSE ranking on held-out test set (time split)				
Model	CYP3A4 (1W0G)	CYP2C8 (2NNI)	CYP2C9 (4NZ2)	CYP2D6 (3QM4)

Baseline	0.4041 + 0.0026	0.4641 + 0.0363	0.4321 + 0.0011	1.2772 + 0.0076
GNN	0.3645 + 0.0011	0.4336 + 0.0070	0.3908 + 0.0037	**1.1668 + 0.0123**
GNN-dummy	**0.3625 + 0.0027**	**0.4238 + 0.0120**	**0.3875 + 0.0054**	1.1704 + 0.0183

**Table 6:** MSE values obtained for compound ranking on the basis of the docking score change for free enzymes for validation and held-out test sets. The lowest MSE values (referring to the lowest error and highest prediction accuracy) are depicted in bold.

MSE ranking on validation test set (random split)				
Model	CYP3A4 (1W0E)	CYP2C8 (1PQ2)	CYP2C9 (1OG2)	CYP2D6 (2F9Q)
Baseline	0.9428 + 0.1226	0.3618 + 0.0227	0.5409 + 0.0703	1.1949 + 0.2464
GNN	0.8814 + 0.1311	0.3218 + 0.0225	0.4856 + 0.0711	1.0591 + 0.2759
GNN-dummy	**0.8699 + 0.1492**	**0.3184 + 0.0189**	**0.4457 + 0.0602**	**1.0521 + 0.2697**

MSE ranking on validation test set (time split)				
Model	CYP3A4 (1W0E)	CYP2C8 (1PQ2)	CYP2C9 (1OG2)	CYP2D6 (2F9Q)

Baseline	0.9488 + 0.3099	0.3374 + 0.0613	0.5361 + 0.2050	1.1274 + 0.7151
GNN	0.9005 + 0.3110	0.3006 + 0.0571	**0.5019 + 0.2000**	1.0438 + 0.7279
GNN-dummy	**0.8878 + 0.3147**	**0.2914 + 0.0411**	0.5024 + 0.2039	**1.0389 + 0.7252**

MSE ranking on held-out test set (random split)				
Model	CYP3A4 (1W0E)	CYP2C8 (1PQ2)	CYP2C9 (1OG2)	CYP2D6 (2F9Q)

Baseline	1.2448 + 0.0016	0.4075 + 0.0144	0.7097 + 0.0108	0.7586 + 0.0010
GNN	**1.1546 + 0.0255**	**0.3940 + 0.0071**	**0.6964 + 0.0136**	0.6513 + 0.0102
GNN-dummy	1.1685 + 0.0226	0.4116 + 0.0122	0.7172 + 0.0211	**0.6476 + 0.0057**

MSE ranking on held-out test set (time split)				
Model	CYP3A4 (1W0E)	CYP2C8 (1PQ2)	CYP2C9 (1OG2)	CYP2D6 (2F9Q)

Baseline	0.5363 + 0.0054	0.4055 + 0.0028	0.3251 + 0.0029	0.6065 + 0.0037
GNN	**0.4912 + 0.0046**	**0.3794 + 0.0030**	0.2829 + 0.0045	**0.5200 + 0.0054**
GNN-dummy	0.4935 + 0.0075	0.4289 + 0.0629	**0.2790 + 0.0042**	0.5308 + 0.0084

In addition, we compare the GNN models using the sum of ranking differences (SRD) [57], [58], [59] – GNN and GNN-dummy achieve comparable performance in compound ranking.

**Fig. 9:**
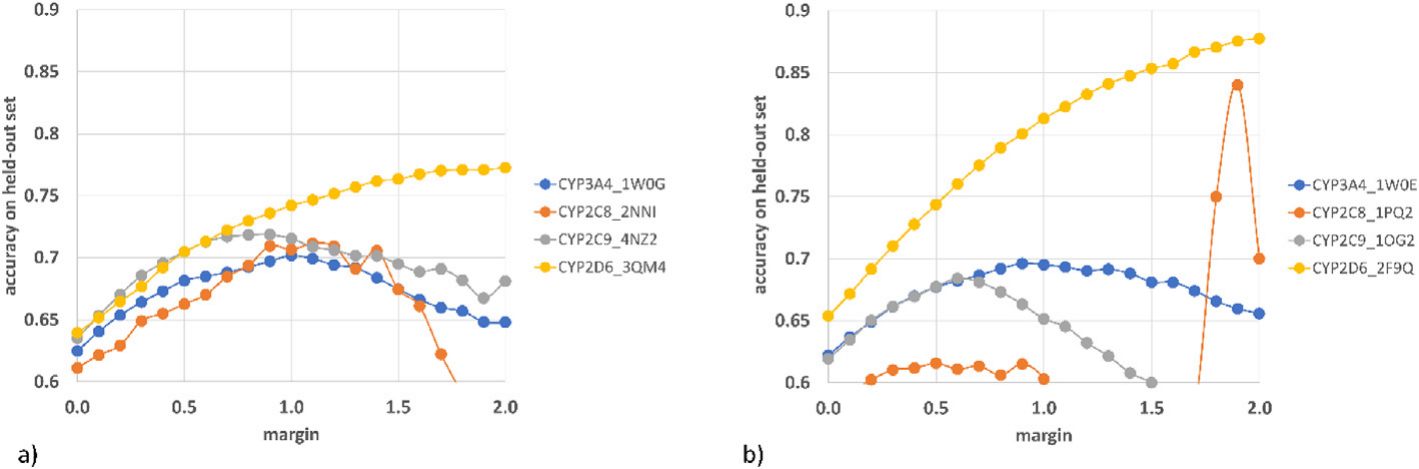
Analysis of changes in the accuracy values for GNN when cases with small changes in the docking score (margin) are neglected, a) crystal structures with inhibitor co-crystallized, b) free crystal structure.

As an additional evaluation, we determine the prediction accuracy of GNN models when cases with very small changes in the docking score (defined as margin) are neglected. We calculate accuracy on held-out test sets with several different values of the margin and present the results in Fig. 9 (the complete data for validation and held-out test sets is present in the Supplementary Data, Fig. S3).

The data presented in Fig. 9 indicates, that negligence of compounds with very small changes in the docking score leads to higher values of accuracy. It is an intuitive outcome, as occurrence of small docking score changes might be to some extent a result of randomness in the docking process and therefore the assignment of a substitution as leading to increase or decrease in the docking score might be biased. For CYP3A4, CYP2C8, and CYP2C9, for crystal structures co-crystallized with respective inhibitors, the accuracy values continuously increase as the margin is getting higher, up to approximately 1, and then, the accuracy drops again. However, it is worth noticing, that at the margin of 2, the accuracy still adopts higher values than when no data is neglected. On the other hand, for 3QM4 (CYP2D6 crystal structure), the accuracy values are constantly rising with the margin increase, reaching a plateau when the margin is equal to approximately 1.7. For free crystal structures, the situation is similar for CYP3A4, CYP2C9 and CYP2D6 (in comparison to inhibited proteins); however, when it comes to the CYP2C8, the accuracy adopts values between 0.60 and 0.62 until the margin of 1.0, whereas further margin increase leads to significant accuracy rise (with values approaching 0.85). It has to be however pointed out that the accuracy values on CYP2C8 might vary more than in the case of other targets, as there is a relatively low number of records in the dataset referring to this cytochrome subtype. This further entails the relatively small held-out test for CYP2C8, which therefore can be biased and can lead to higher variation in the results. Overall, this results suggest, that GNN models more often predict the docking score change correctly if its value is high enough. This indicates that, in some cases, incorrect prediction of the docking score change can be attributed to randomness of the docking score acquisition.

### 3.5 Saliency maps help designing new CYP inhibitors

In this section, we show how saliency maps can be used to enable guidance for the process of designing new CYP inhibitors. Saliency maps show which structural features of the input compound influence the obtained predictions to the highest extent.

**Fig. 10:**
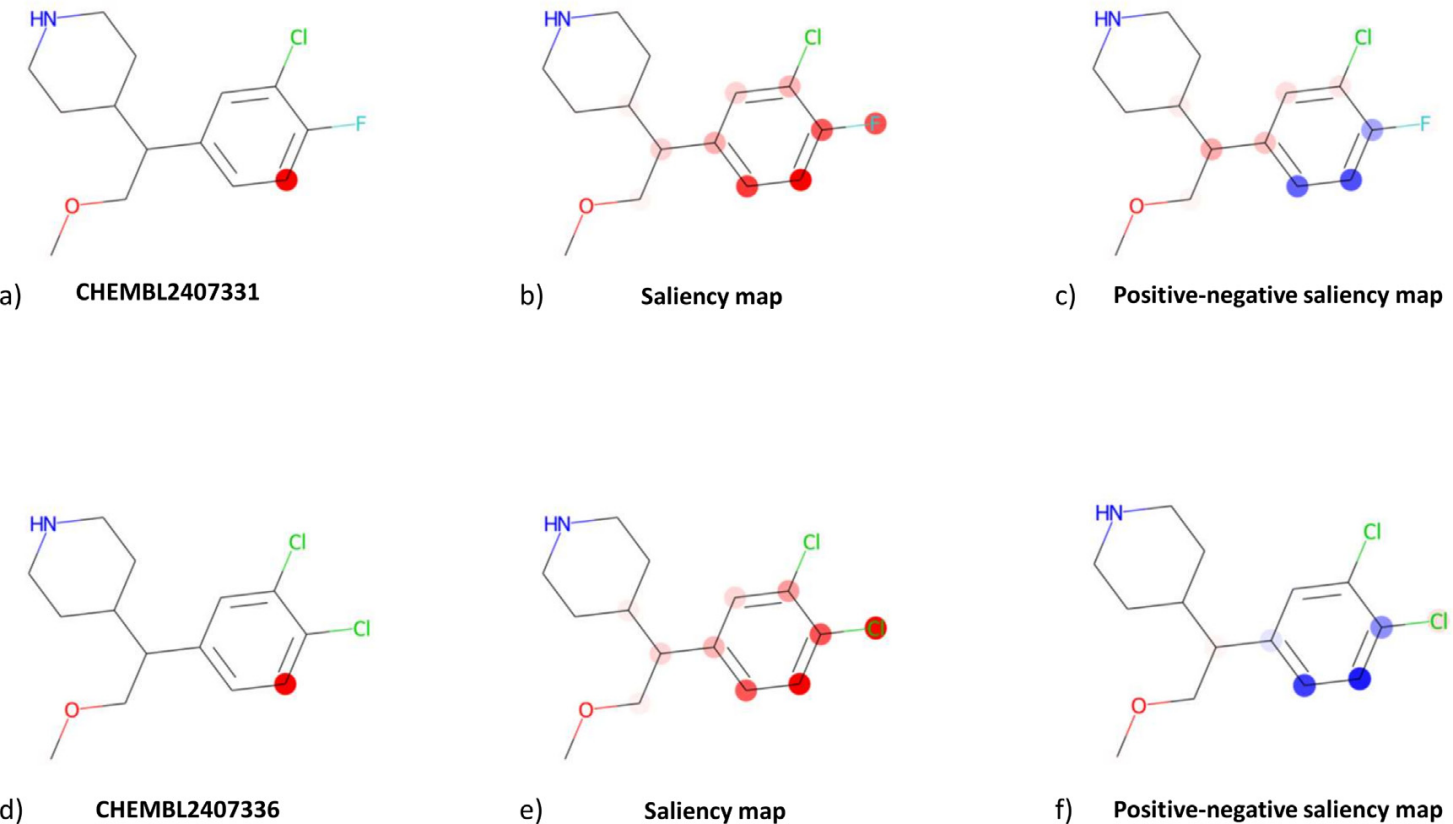
Outcome of the explainability analysis for the selected compounds, a) indication of the atom in the CHEMBL2407331 structure for which the predictions are analyzed, b) saliency map for CHEMBL2407331, c) positive -- negative saliency map for CHEMBL2407331 (difference between the classical saliency map and “inverse” saliency map calculated on the negative partial derivatives), d) indication of the atom in the CHEMBL2407336 structure for which the predictions are analyzed, e) saliency map for CHEMBL2407336, f) positive -- negative saliency map for CHEMBL2407336.

In Fig. 10, we present saliency maps obtained for CHEMBL2407331 (top row) and CHEMBL2407336 (bottom row). In both cases the explanations are given for prediction of a docking score change of compounds modified by attaching fluorine to the atom marked with a red dot in leftmost pictures. The presented molecules differ by only one atom (fluorine in CHEMBL2407331 is substituted with chlorine in CHEMBL2407336) and their explanations are similar. However, a few differences can be spotted. In the presented saliency maps saturation represents the value, with lighter shades marking values closer to zero, and color represents the sign – red for positive and blue for negative values.

At the first glance, one can observe that the only atoms identified as important are these in the close proximity of the atom for which the prediction is made. This stems directly from the definition of saliency maps and the fact that the model being explained has 5 convolutional layers. The number of convolutional layers restricts the neighbourhood from which information can be utilised to make a prediction. As a result, the outcome is insensitive to information from outside of this neighbourhood, and thus the respective partial derivatives are equal to zero.

Another similarity is the relative importance of the atoms, which can be partly attributed to the fact that the closer an atom is to the one for which a prediction is made, the more times its information is used to calculate this prediction. Furthermore, in case of positive–negative saliency maps the sign of the calculated importance is an another similarity as blue dots and red dots are similarly distributed.

For both molecules classical saliency maps indicate that the halogen atom that is closer to the substituted atom is more important than the other one. On the other side, positive–negative saliency map for CHEMBL2407331 (Fig. 10c) assigns higher importance to the halogen further away. This indicates that the negative saliency map assigns a higher value to this atom then the classical saliency map, whereas the values for the other halogen are similar for both classical and negative saliency maps. Intuitively, this means that the value of the prediction is sensitive to information encoded in both halogens; however, in the case of chlorine negative partial derivatives dominate while in the case of fluorine the positive and negative partial derivatives cancel each other out. In the case of CHEMBL2407336 the situation is reversed. These findings might suggest that the modified molecules take slightly different poses in the binding pocket, and thus different atoms are more important for the ligand-CYP interactions.

### 3.6 Visualization tool

**Fig. 11:**
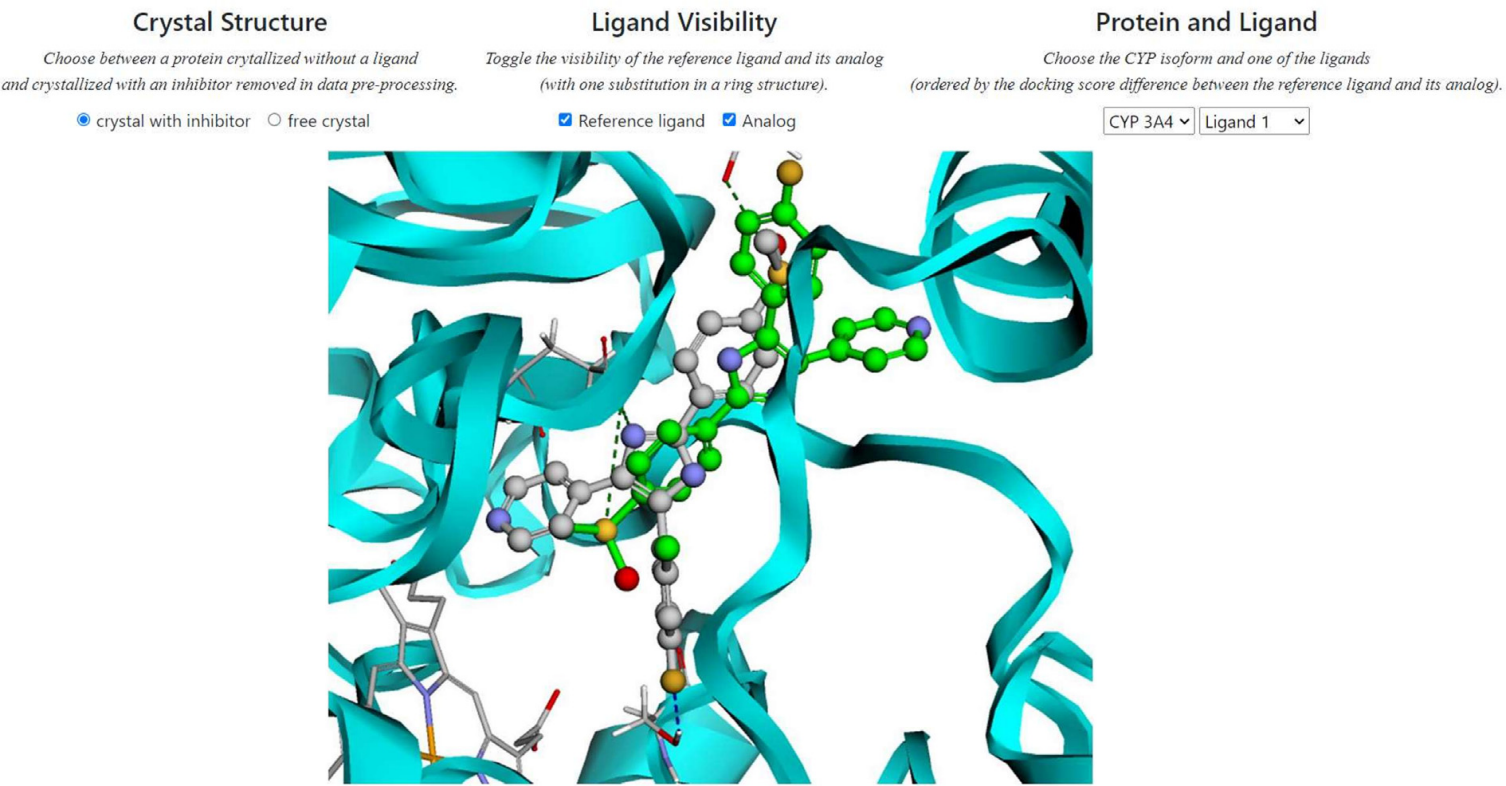
Visualization of the docking poses obtained for known CYP3A4 inhibitor (CHEMBL10, CYP3A4 inhibition: 61%, depicted in green) and its derivative (chlorine substitution, depicted in gray). (For interpretation of the references to color in this figure legend, the reader is referred to the web version of this article.)

We developed a visualization platform in order to enable a manual inspection of the docking poses obtained for the generated compounds and their comparison with the ligand–protein complexes for the existing inhibitors. The platform is available at https://gmum.github.io/cyp-inhibitors/. It enables instant confrontation of the obtained changes in the docking scores (original compound *vs* derivative) with the actual compound docking poses (Fig. 11).

## 4 Conclusions

In the study, we develop a protocol for generation of new inhibitors of selected CYPs. Although, the procedure is optimized for this particular target, it can be applied to any protein in a similar manner, as the code is available and includes all scripts required to reproduce the results (https://github.com/gmum/cyp-inhibitors).

The subsequent stages of the proposed methodology are composed of generation of new derivatives of known CYP inhibitors, docking and evaluation of the compound possible inhibition on the basis of the obtained ligand–protein complexes. Moreover, an innovative GNN model for prediction of the docking score change upon particular substitution is proposed. This model makes predictions for particular graph vertices, not a compound graph as a whole. The activity predictions obtained for the generated compounds can be analyzed in detail using saliency maps to detect structural features, which influence to the highest extent the predictions of the inhibitory potency of the newly formed molecules. Our data suggests that even a single substitution can lead to a huge difference in the docking score value; however, in most cases this difference is negligible. Furthermore, using more than a single substitution does not seem to further improve the docking score.

Moreover, we prepared a visualization platform (https://gmum.github.io/cyp-inhibitors/), where the docking poses of the newly formed inhibitors can be manually inspected and confronted with the docking outcome of the original compounds. The results not only provide a library of new potential CYP inhibitors (all generated compounds are shared in the Supplementary Data), but can also guide the process of designing new compounds with CYP inhibitory properties. Availability of all scripts used in the study (at https://github.com/gmum/cyp-inhibitors) makes the developed tools general and enable their application for any target.

## Funding

The study was supported by the grant OPUS 2018/31/B/NZ2/00165 financed by the National Science Centre, Poland (www.ncn.gov.pl). The work of TD was supported by the grant PRELUDIUM 2020/37/N/ST6/02728 financed by the National Science Centre, Poland (www.ncn.gov.pl). This research was supported in part by PL-Grid Infrastructure.

## CRediT authorship contribution statement

**Tomasz Danel:** Investigation, Data curation, Methodology, Software, Visualization, Writing – review & editing, Funding acquisition. **Agnieszka Wojtuch:** Investigation, Methodology, Visualization, Writing – review & editing. **Sabina Podlewska:** Conceptualization, Formal analysis, Supervision, Writing – original draft, Funding acquisition.

## Declaration of Competing Interest

The authors declare that they have no known competing financial interests or personal relationships that could have appeared to influence the work reported in this paper.

## Appendix A Supplementary data

Supplementary data 1

Supplementary data 2 Library of new potential CYP inhibitors generated separately for each crystal structure considered in the study. Within each file, the compounds are sorted according to the decreasing difference in the docking score values between the generated compound and the original structure.
