# Supplementary material for: Generation of new inhibitors of selected cytochrome P450 subtypes– In silico study
Source: Comput Struct Biotechnol J. 2022 Oct 6;20:5639–51. doi: 10.1016/j.csbj.2022.10.005 (PMC9582735; doi:10.1016/j.csbj.2022.10.005)
Supplement: Supplementary data 1 [file mmc1.pdf]

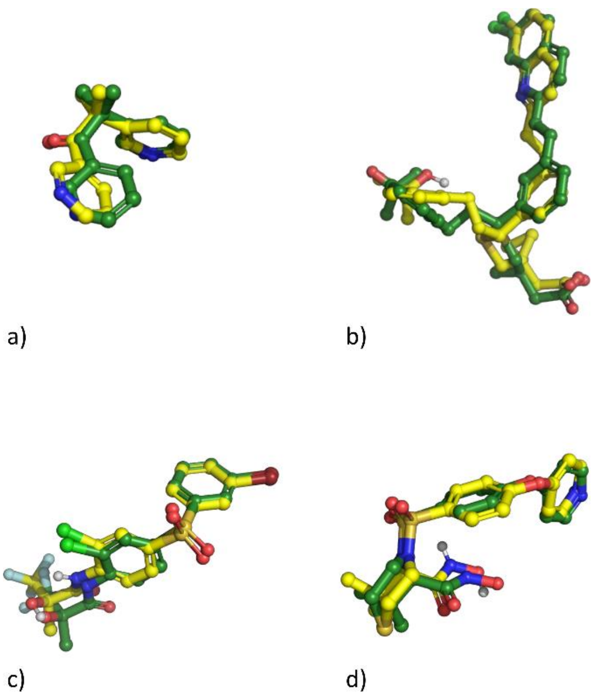

Figure S1. Visualization of the results of the procedure of redocking CYP inhibitors present in the respective crystal structures; green – co-crystallized conformation; yellow – conformation obtained in docking, a) CYP3A4, b) CYP2C8, c) CYP2C9, d) CYP2D6.

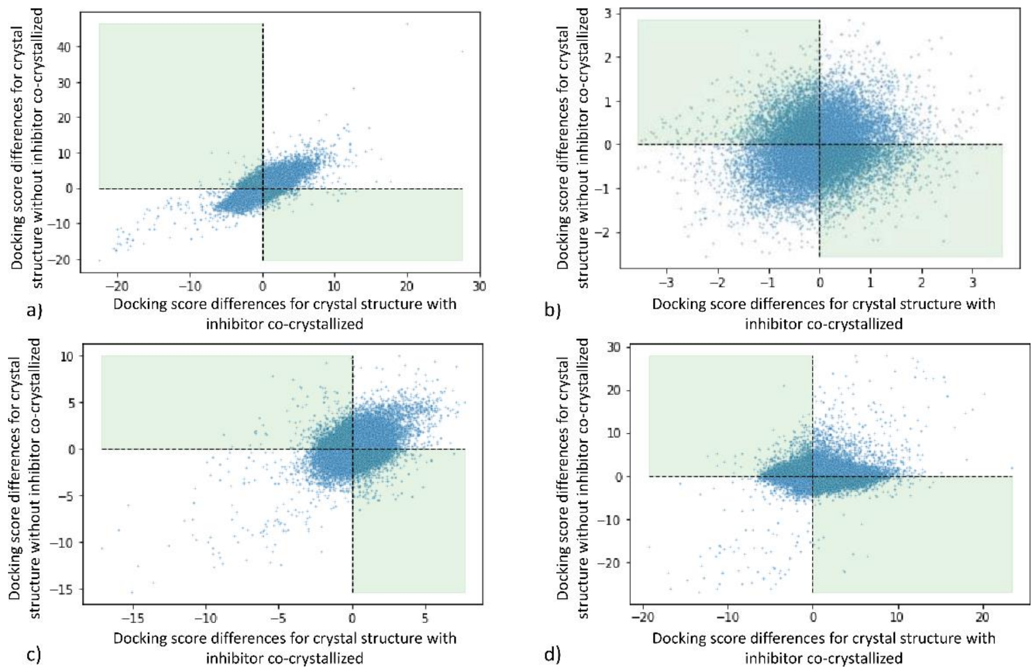

Figure S2. Examination of differences in changes in the compound docking scores upon substitution between free crystal structures and those with the respective inhibitors co-crystallized for a) CYP3A4, b) CYP2C8, c) CYP2C9, d) CYP2D6.

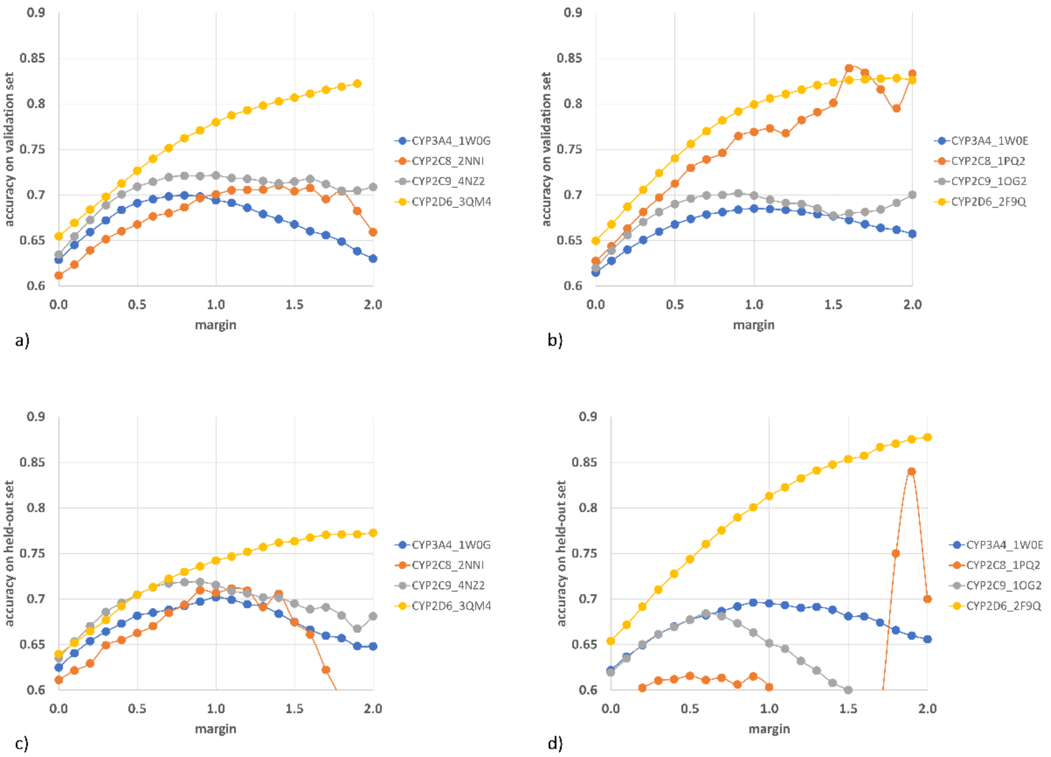

Figure S3. Analysis of changes in accuracies of predicting the sign of docking score change for different thresholds for cases negligence (margin), a) validation sets for crystal structures co-crystallized with inhibitor, b) validation sets for free crystal structures, c) held-out test sets for crystal structures co-crystallized with inhibitor, d) held-out test sets for free crystal structures.
